# Identification of actin cytoskeleton organization genes in oral cancer and oral potentially malignant disorders using oral tissue RNA-seq database

**DOI:** 10.4317/medoral.27364

**Published:** 2025-08-16

**Authors:** Marta Serna-García, Agnese Formaggio, María Carmen Carceller, Joaquín Javier Panadero Romero, Nicla Flacco

**Affiliations:** 1 European University of Valencia, Faculty of Health Sciences, Department of Dentistry, Valencia, Spain; 2 Department of Pharmacy, Pharmaceutical Technology and Parasitology, Faculty of Pharmacy and Food Sciences, University of Valencia, Burjassot, Valencia, Spain.; 3 Interuniversity Research Institute for Molecular Recognition and Technological Development (IDM), University of Valencia, Polytechnic University of Valencia, Burjassot, Valencia, Spain; 4 Igenomix S.L., Valencia, Spain

## Abstract

**Background:**

Oral cancer and oral potentially malignant disorders (leukoplakia and oral submucous fibrosis) are prevalent and clinically significant oral diseases. Actin, crucial for epithelial tissue integrity, undergoes cytoskeleton reorganization associated with increased invasiveness in oral cancer.

**Material and Methods:**

Bioinformatic analysis of RNA-seq data from GEO public databases was performed to detect differentially expressed genes in oral cancer, leukoplakia and oral submucous fibrosis. Enrichment analysis of the differentially expressed genes was performed using DAVID and GSEA software. ROC curve and survival analysis were conducted to assess the discriminative capacity of these genes as possible biomarkers. The results were further validated using RNAseq data from The Cancer Genome Atlas (TCGA).

**Results:**

EPRS1 was consistently overexpressed in all three pathologies. Key genes (ACTIN1, LIMK1, CORO1C, INF2, SH3D21, CFL1, FSCN1, MYO1B) implicated in actin cytoskeleton organization were identified, suggesting their role in oral potentially malignant disorders and cancer progression. Receiver operating characteristic (ROC) curves on 522 TCGA samples demonstrated these genes' potential as early biomarkers for oral cancer, with their inhibition associated with improved survival.

**Conclusions:**

The identified genes offer insights into actin-related mechanisms and potential pathways for the diagnosis and treatment of oral cancer. Nonetheless, further research is essential to validate these results.

** Key words:**RNA-seq, oral cancer, oral submucous fibrosis, leukoplakia, actin.

## Introduction

Oral cancer and oral potentially malignant disorders involve alterations in the oral epithelial tissue, characterized by morphological and functional changes in the cytoskeleton. Actin, a crucial protein in cytoskeletal organization, plays a key role in cellular processes such as adhesion, migration, and proliferation. In epithelial cells, actin forms intracellular filaments that provide structural support and contribute to cellular shape and contraction (1).

Actin microfilaments are essential for the biogenesis and function of the apical junctional complex (AJC). Remodeling of apical junctions requires significant reorganization of the perijunctional F-actin ring. Disruption of microfilaments prevents AJC assembly by eliminating AJC proteins from cell-cell contact areas. Similarly, inactivation or hyperactivation of Rho family GTPases, which modulate F-actin architecture, can disrupt apical junctions. Genetic removal or overexpression of actin-binding proteins lacking actin-binding sites also hinders the formation of epithelial AJCs (2). Anomalous actin reorganization in epithelial tissue is linked to malignant traits and loss of normal cellular architecture. Epithelial-Mesenchymal Transition (EMT) enables the transition between epithelial and mesenchymal states, altering cell polarity, cytoskeletal organization, signaling, and gene expression, promoting cell motility and invasiveness. EMT plays a vital role in development, wound healing, fibrosis, and cancer progression (3).

Studies have demonstrated the involvement of actin and its regulation in oral carcinogenesis and the progression of oral cancer. Notably, the reorganization of the actin cytoskeleton has been linked to the acquisition of a more invasive phenotype in cancer cells (4). Given that the expression of cytoskeletal proteins is directly correlated with the degree of malignancy, an understanding of these proteins offers valuable tools to enhance cancer prognosis and treatment (5). Studying actin-related mechanisms can open new opportunities for research and the development of therapeutic and diagnostic strategies. Precancerous conditions and oral cancer can have a devastating impact on patients' quality of life, and early detection and accurate diagnosis are fundamental to successful treatment.

Recent advances in scientific research, facilitated by technologies like RNA-seq, have offered deeper insights into the molecular aspects of oral diseases. Despite the complexity and heterogeneity of these conditions, analyzing gene expression profiles using consolidated datasets from databases like The Cancer Genome Atlas (TCGA) can identify potential biomarkers and pave the way for early and precise diagnosis, as well as the development of tailored therapies.

This study aims to explore the gene expression profile in oral cancer, focusing on genes related to actin cytoskeleton organization and those commonly implicated in both cancer and precancerous diseases. Such investigations are pivotal for enhancing our understanding of the molecular underpinnings of oral epithelial tissue dysfunctions, particularly in relation to cancer and precancerous states, thereby advancing the search for early indicators of oral cancer.

Abbreviations used throughout the manuscript include:

ADF (actin-depolymerizing factor), AJC (apical junctional complex), C (cancer), DEGs (differentially expressed genes), EMT (epithelial-mesenchymal transition), F (fibrosis), FDR (false discovery rate), GEO (Gene Expression Omnibus), GSEA (Gene Set Enrichment Analysis), KEGG (Kyoto Encyclopedia of Genes and Genomes), L (leukoplakia), N (non-pathology), NCBI (National Center for Biotechnology Information), OSCC (oral squamous cell carcinoma), OSF (oral submucous fibrosis), RNA-seq (RNA sequencing), and TCGA (The Cancer Genome Atlas).

## Material and Methods

Three RNA-seq datasets were included in our study (6,7,8). These datasets were obtained from the Gene Expression Omnibus (GEO), which is hosted by the National Center for Biotechnology Information (NCBI) and is a publicly available genomics database that collects submitted high-throughput gene expression data (https://www.ncbi.nlm.nih.gov/geo/). Bioinformatic analysis was performed for the detection of differentially expressed genes in oral cancer (C) and two oral potentially malignant disorders, such as leukoplakia (L) and oral submucous fibrosis (F), compared to non-pathology (N) samples, as summarized in Table 1.

In order to corroborate the veracity of our findings within the realm of oral cancer, comprehensive comparisons were undertaken utilizing the TCGA oral cancer database. This validation process served to bolster the overall robustness of our research outcomes. Gene expression data, in conjunction with the corresponding clinical data pertaining to the Head and Neck Squamous Cell Carcinoma samples, were meticulously acquired from the TCGA data portal, accessible at https://portal.gdc.cancer.gov.

- Differential expression analysis

Four differential expression analyses were performed using the raw data from patient samples obtained from the selected three studies (Table 1).

All raw files corresponding to each sample from the studies were analyzed using the same workflow. The quality of RNA-Seq reads (quality scores, GC content, N content, length distributions, duplication levels, overrepresented sequences, and K-mer content) was assessed using Fastqc software v.0.11.4.

Low-quality reads (adapter sequence and reads containing poly-N) from the raw data were removed using Trimmomatic v.0.36 with the following parameters: java -jar /usr/local/bin/trimmomatic-0.36.jar PE -phred33 <IN_R1.fastq.gz> <IN_R2.fastq.gz> <OUT_R1.fastq.gz> <OUT_R1_UN.fastq.gz> <OUT_R2.fastq.gz> <OUT_R2_UN.fastq.gz> LEADING:3 TRAILING:3 SLIDINGWINDOW:4:15 MINLEN:50.

All subsequent analysis was based on the high-quality trimmed data. We used Kallisto software v.12 to align the trimmed paired-end reads to the Homo sapiens reference genome deposited in the National Center for Biotechnology Information (NCBI) (https://www.ncbi.nlm.nih.gov/). Read counting was performed using the internal script and Ensembl annotations (Index: /pub/release-105/gtf/homo sapiens/ (ensembl.org)) for gene annotations.

The EdgeR and Limma packages available from the Bioconductor project were used to estimate normalized counts per million (CPM) and identify differentially expressed genes (DEGs) between the groups. EdgeR was used for data normalization, and the Limma software was used for statistical methods (linear modeling and empirical Bayes), following the methodology described by Law el al. (9). We considered a gene to be differentially expressed when the false discovery rate (FDR) value was ≤ 0.05.

- Enrichment analysis

Enrichment analysis of the differentially expressed genes was performed using the DAVID software v.6.8 (https://david.ncifcrf.gov) with the Kyoto Encyclopedia of Genes and Genomes (KEGG) database (*p value* ≤ 0.05) and Gene Set Enrichment Analysis (GSEA) was performed using GSEAPreranked tool and GSEA function on clusterProfiler package in R.

Visualization of interrelations of terms and functional groups in biological networks was perfomed using ClueGO plug-in of Cytoscape software v3.6.0.

- Validation using the TCGA dataset

RNA sequencing data (RNA-Seq) and corresponding clinical data for Head and Neck Squamous Cell Carcinoma samples were downloaded from the TCGA data portal (https://portal.gdc.cancer.gov), specifically from the Head and Neck Squamous Cell Carcinoma project (TCGA, PanCancer Atlas). The inclusion criteria for samples were as follows: (i) gene expression profiles must be available in the dataset; (ii) clinical data for patients, including gender, age, and overall survival status data, were required. A total of 522 patients were ultimately included in the study. To obtain the predictors or biomarkers, receiver operating characteristic curves (ROC) were generated. For the analysis, the genefilters package (Bioconductor) was used with the used function of “rowpAUCs”. This function was applied to the normalized values (TPMs) in the 522 TCGA samples. An area under the ROC curve (AUC) of 0.5 indicates a test with no discriminatory ability, essentially performing no better than chance. Conversely, an AUC of 1.0 signifies a test with perfect discrimination (10).

Survival analysis was performed using the survival package v.3.2-7 (Bioconductor), based on clinical data and gene expression profiles obtained from TCGA (PanCancer Atlas, 522 samples). Patients were stratified into high (Up) and low (Down) expression groups using the mean gene expression value as the threshold. Custom R scripts were used to process gene expression data and generate Kaplan-Meier survival curves, based on overall survival status and follow-up time provided in the clinical manifest.

## Results

- Differentially Expressed Genes

The human gene set, Homo_sapiens.GRCh38.96.gtf, which includes a total of 58,884 coding and non-coding genes, was used for expression quantification. When analysing the cancer’s samples (C vs N), 1,408 DEGs (800 underexpressed genes and 608 overexpressed genes) were identified from study_1 (GSE125866), and 8 DEGs (4 underexpressed genes and 4 overexpressed genes) were found from study_2 (GSE20116). Regarding the oral potentially malignant disorders, 110 DEGs (102 underexpressed genes and 8 overexpressed genes) were found in F vs N (GSE125866) analysis, while 5,170 DEGs (2,567 underexpressed genes and 2,603 overexpressed genes) were identified in L vs N (GSE131568) analysis.

All the obtained differentially expressed genes were subjected to FDR correction <0.05 (Supplement 1, available at Zenodo: https://zenodo.org/records/15338531).

- Common genes in different pathologies

Oral cancer and leukoplakia shared 154 common overexpressed DEGs (Supplement 2) and 105 common underexpressed DEGs. There were 4 common overexpressed DEGs (Supplement 3) and 43 common underexpressed DEGs between oral cancer and oral submucous fibrosis. However, oral submucous fibrosis and leukoplakia had 1 common overexpressed gene (PTPN11) and 1 common underexpressed gene (ST7L). The three pathologies shared only a common overexpressed gene, but any underexpressed ones. The unique gene that was found in all cancer and oral potentially malignant disorders was EPRS (glutamyl-prolyl-tRNA synthetase 1), with a fold change of 0.653 (C vs N), 0.492 (F vs N) and 0.091 (L vs N) (Supplement 4).

- Enrichment analysis

An enrichment analysis of the 160 common overexpressed DEGs among at least two of the three pathologies was performed using the DAVID software with a *p value* less than 0.05 (Supplement 5).

**Figure 1:**
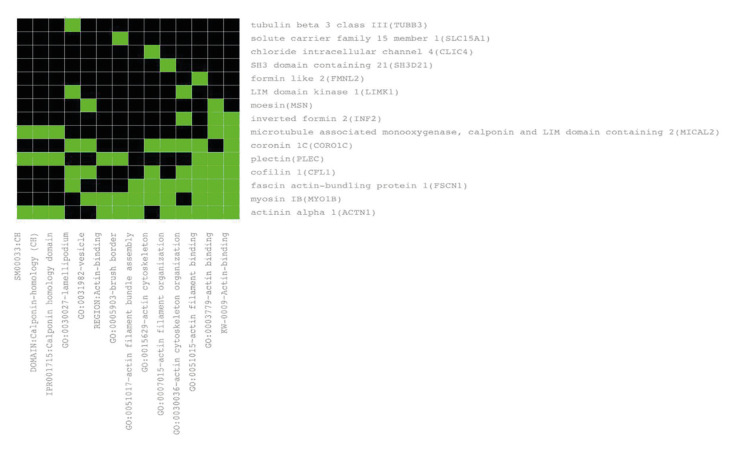
Correlations between genes with biological function and actin related processes, using DAVID software.

**Figure 2:**
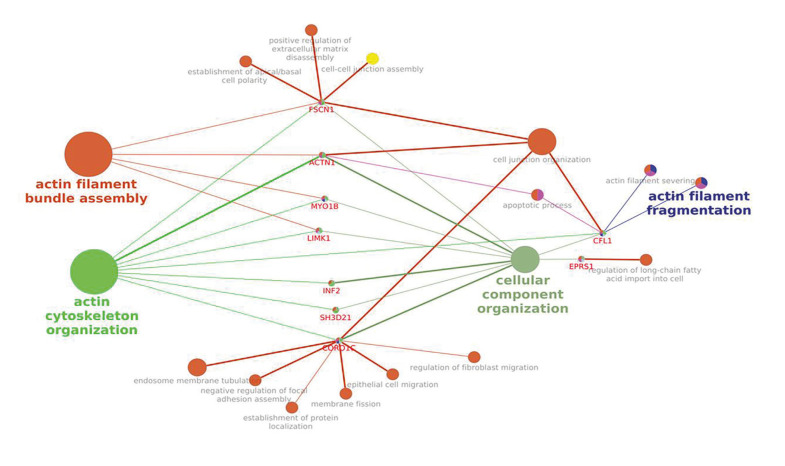
Interaction networks between common overexpressed DEGs EPRS1, ACTN1, INF2, CFL1, LIMK1, FSCN1, MYO1B, SH3D21, and CORO1C and their biological processes using Cytoscape software.

Among the genes which play biological functions, 8 genes including INF2, ACTN1, CFL1, LIMK1, FSCN1, MYO1B, SH3D21 and CORO1C, highlighted to be specially involved in different actin processes, such as actin filament organization, actin filament bundle assembly and actin cytoskeleton organization (Fig. 1, Fig. 2).

**Figure 3:**
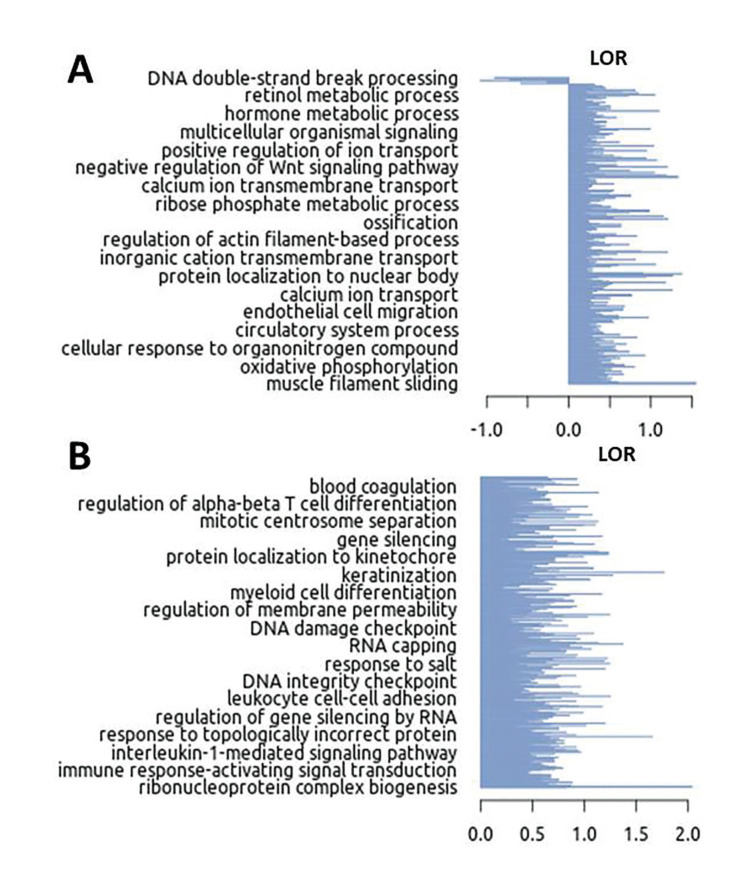
Significant biological processes in leukoplakia (L) samples (A) and oral cancer (C) samples (B) using GSEA. The GSEA software used the LOR (Logarithm of Ratio) refer to L and C group, this value categorizes each biological process in a classification in relation to the rest (total), allowing to identify biological processes, molecular mechanisms and significant cellular components and their regulation within the groups.

Finally, we obtained the most relevant biological processes of all genes with expression changes of C vs N and L vs N using the GSEA software. We observed that both C and L samples had processes of interest in the study, such as endothelial cell migration, regulation of actin filaments-based process, and keratinization activation (Fig. 3).

- Validation of Markers Using TCGA Data

**Figure 4:**
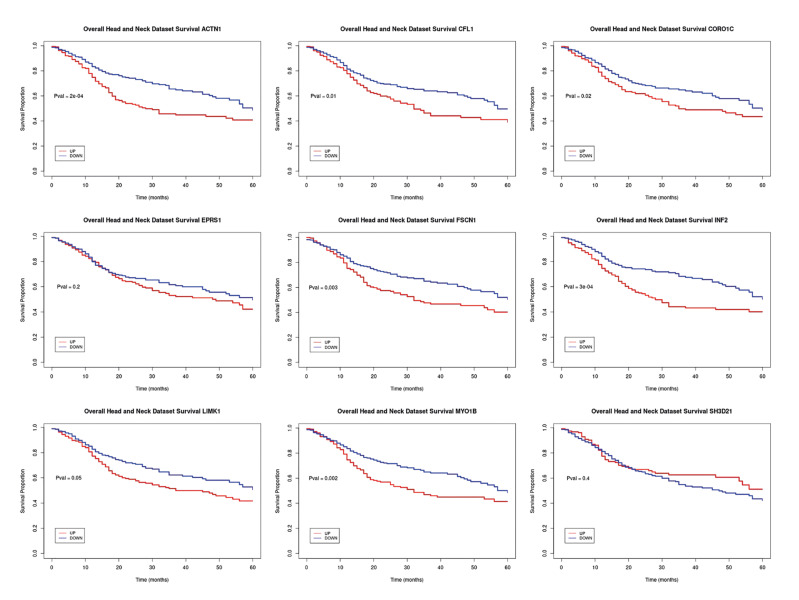
Kaplan-Meier survival curves of the 9 marker genes in 522 TCGA samples, demonstrating that the overexpression of these genes correlates with poorer survival in HNSCC.14/6/2025

This validates the potential for these genes to serve as early indicators in the future. Furthermore, in the analysis of ROC curves, we identified nine biomarkers from 522 TCGA samples. Interestingly, eight genes stood out as genes of interest in our study. Notably, six of these genes had an area under the curve greater than 0.79: ACTN1 (0.96), MYO1B (0.91), FSCN1 (0.89), INF2 (0.86), CORO1C (0.81), and LIMK1 (0.79), indicating their potential as robust markers. In the survival analysis of these genes in the 522 oral cancer samples, we observed similar trends across all cases, with statistically significant results (*p* ≤ 0.05) for most markers, except for two of them (EPRS1 and SH3D21). We found that high expression of these markers correlated with poorer survival rates, as shown in Fig. 4.

## Discussion

The gene EPRS1 (Glutamyl-prolyl-tRNA synthetase 1) is considered the most relevant in our study as it is found to be overexpressed in all samples associated with oral cancer, oral submucous fibrosis, and oral leukoplakia. EPRS1 encodes for the protein glutamyl-prolyl-tRNA synthetase 1, a member of the aminoacyl-tRNA synthetase (AaRS) family. AaRS plays a fundamental role in protein synthesis by pairing tRNA with their corresponding amino acids to decode mRNA based on the genetic code (11).

Recent studies have suggested that EPRS1 may play possible roles in the progression of liver cancer, as it promotes the synthesis of gene sets associated with metastasis (hallmark epithelial-mesenchymal transition). Considering this, EPRS1 may serve as a potential treatment target, its overexpression predicts poor prognosis in gastric cancer patients. Also identified gains in the copy number of EPRS1 in lung, esophageal, hepatocellular, skin, and breast tumors (12).

EPRS1 has been identified as a positive regulator of collagen levels, a key component of the extracellular matrix. EPRS1 is expressed in the basal layers of the skin epithelium, and its expression is correlated with the differentiation and barrier function of the epithelial tissue. Moreover, overexpression of EPRS1 in human keratinocyte cells promotes differentiation and the expression of barrier function markers in the skin epithelial tissue (https://www.proteinatlas.org/ENSG00000136628-EPRS1). EPRS1 activation has been implicated in renal fibrosis by promoting fibroblast activation and mitochondrial dysfunction, which in turn drive extracellular matrix accumulation and contribute to disease progression (13).

In oral squamous cell carcinoma (OSCC), suppression of EPRS1 expression in tumor cells has been shown to decrease cell proliferation, indicating a potential functional role for EPRS1 in OSCC development (14). Accordingly, its overexpression in certain cancer types may influence both cell proliferation and tumor progression. However, further research is required to elucidate the underlying mechanisms and define the precise role of EPRS1 in epithelial tissue.

Among the overexpressed genes that are most involved in the orchestration of the actin cytoskeleton, ACTN1, also known as "non-muscle actinins," plays a significant role in regulating cytokinesis, cellular adhesion, migration, and mediating sarcomere function. Studies suggest that elevated ACTN1 expression destabilizes E-cadherin-based adhesions, potentially enhancing the migratory capacity of breast cancer cells (15).

In our analysis, we observed the overexpression of this gene in patients with oral cancer, which is consistent with the study by Guo-Feng Xie. They considered ACTN1 as one of the primary markers in tissues affected by OSCC. Additionally, elevated levels were associated with invasion, metastasis and a poor prognosis (16). The identification of these genes as potential prognostic biomarkers and their implications in promoting cancer cell migration and metastasis warrant further investigation and could have important implications for cancer diagnosis and treatment strategies.

Another overexpressed gene that has been found relevant in our study is LIMK1 (LIM Kinase 1), a serine kinase that catalyzes the phosphorylation of the hydroxyl group of serine, which regulates actin polymerization and the depolymerization of microtubules (17).

In the context of tumor cell invasion and metastasis, various pseudopods, including filopodia, are formed. These dynamic structures are dependent on continuous actin polymerization and depolymerization processes, which can be influenced by various factors. Among these factors, LIMK1 emerges as a critical regulator of cytoskeletal remodelling. LIMK1 controls the activity of actin-depolymerizing factor (ADF), also known as destrin / cofilin, a family of actin-binding proteins that play a significant role in filopodia formation (18). These findings underscore the importance of actin dynamics and LIMK-mediated cytoskeletal regulation in the metastatic process. According to our study, LIMK1 has been found to be overexpressed in OSCC. This observation is consistent with other studies that indicate the involvement of LIMK1 in the tumorigenesis and metastasis of oral cancer (19).

An additional overexpressed gene that has demonstrated importance in our study is COROC1 (Coronin 1C), which encodes an actin-binding protein and belongs to the coronin family, known for their role as linkers between actin and other proteins in the cellular cytoskeleton. Coronins are a conserved family of actin cytoskeleton regulators that promote cell motility and modulate other actin-dependent processes. Furthermore, COROC1 is involved in the dynamic regulation of actin networks and cellular motility related to microfilaments. CORO1C is localized in areas where dynamic changes of cellular microfilaments are active, such as lamellipodia or membrane ruffles. Additionally, CORO1C has been demonstrated to be involved in the tumorigenesis and metastasis of OSCC where it is found to be overexpressed (20).

The gene INF2 (Inverted Formin 2) encodes a protein that participates in both actin filament polymerization and depolymerization. INF2 has been implicated in the formation of invadopodia, specialized structures that facilitate cell migration and invasion (21). In our study, we found INF2 to be overexpressed in both oral leukoplakia and oral cancer. It was also found to be overexpressed in basal-type breast cancer. Additionally, a study indicated that the elimination of INF2 could reduce tumor cell proliferation (22).

We also observed an elevated activity of the gene SH3D21, which is involved in actin filament organization and cell migration. This gene has been considered as potentially related to the activation and sensitization of cancer cells, earning the designation of a survival gene for lung cancer (23).

The CFL1 gene (Cofilin 1) is a key regulator of actin cytoskeletal dynamics. The cofilin pathway has emerged as playing a central role in cytoskeletal reorganization, mediating actin filament remodeling through polymerization and depolymerization. Filament remodeling is essential during the formation and retraction of pathfinding structures used in chemotaxis, cell migration, and tumor cell invasion (24).

In the context of advanced epithelial ovarian cancer, it was observed that patients with low-expression of CFL1 had longer progression-free survival compared to those with high-expression cases (25). Similarly, in our study, we found elevated levels of the CFL1 gene in the epithelial tissue of the oral cavity in patients with malignant lesions.

Turhani reported high CFL1 expression in OSCC, and they also identified associations with cancers of the pancreas, breast, and gallbladder (26).

FSCN1 is involved in the formation of actin-based structures like filopodia, lamellipodia, and other membrane extensions, which are essential for cell migration and attachment to the extracellular matrix. Studies have reported significant FSCN1 expression in laryngeal squamous cell carcinoma tissues, suggesting its role in the malignant progression of this cancer (27,28). Inhibition of FSCN1 using anticancer drugs has shown potential for cancer treatment and clinical applications, making it a promising therapeutic target for various cancer types (29).

MYO1B is considered important for cell migration and cellular motility. In colorectal cancer, this gene promotes the reorganization of F-actin through the previously mentioned genes LIMK1 and CFL1 by enhancing RhoA activation. MYO1B also promotes focal adhesion assembly by targeting RhoA. In addition, disruption of apical junctions can be achieved by either inactivation or hyperactivation of Rho family GTPases, which are well-known modulators of F-actin architecture (30). Finally, genetic elimination of actin-binding proteins or overexpression of mutant junctional proteins lacking actin-binding sites impairs the formation of the epithelial apical junctional complex (AJC). Given the pivotal role of the actin cytoskeleton in the biogenesis of the epithelial AJC, it is crucial to understand the mechanisms of F-actin reorganization that drive the assembly of intercellular junctions (2).

Recent studies have identified MYO1B as a key gene in oral cancer, with its overexpression being associated with lymph node metastasis and unfavorable outcomes. Suppression of MYO1B can inhibit the proliferation, invasion, and metastasis of oral cancer cells, making it a potential therapeutic target for oral cancer. These findings suggested that MYO1B could serve as a novel prognostic indicator for oral cancer (31).

We have identified two common genes, PTPN11 and ST7L, that are commonly deregulated in the precancerous conditions OSF and oral leukoplakia, despite their distinct etiological origins. Specifically, PTPN11 is overexpressed and ST7L is underexpressed in both conditions, suggesting the involvement of common molecular pathways. While OSF is primarily linked to areca nut chewing and leukoplakia to tobacco and alcohol use, both conditions are characterized by chronic inflammation and dysregulation of cell growth and differentiation. These common biological processes may underlie the observed convergence in gene expression, pointing toward potentially overlapping pathogenic pathways.

PTPN11 (also known as Shp2) plays a key role in cellular development and mediates signaling pathways trigged by growth factors, cytokines, and the extracellular matrix. Its overexpression is associated with fibrosis and tumorigenesis, particularly in OSCC, where it activates RTK pathways like EGFR, PI3K/AKT, and MAPK. This enhances cell proliferation, survival, and evasion of apoptosis, contributing to OSCC and precancerous lesion progression (32,33).

On the other hand, ST7L is involved in the regulation of apoptosis and the cell cycle. Its overexpression has been linked to increased apoptosis and inhibited proliferation in certain cancer types. It may suppress the AKT/GSK3β/β-catenin pathway and act as a tumor suppressor. Conversely, the underexpression of ST7L in precancerous conditions like OSCC may promote apoptosis evasion and progression toward more aggressive cancer forms (34-36).

## Conclusions

Analysing the transcriptome of oral potentially malignant disorders and cancerous diseases could provide insights into cancer progression, shedding light on the underlying biological processes. This illuminates novel avenues for the identification of potential biomarkers. In this study, we analysed public RNA-seq data to identify the differentially expressed genes in oral cancer, leukoplakia and oral submucous fibrosis. We spotlighted eight key genes, including ACTIN1, LIMK1, CORO1C, INF2, SH3D21, CFL1, FSCN1, and MYO1B, which play roles in the intricate organization of the actin cytoskeleton and were overexpressed in the studied oral conditions. Moreover, the gene EPRS1 was the only one overexpressed in all studied diseases, emerging as a candidate for a potential early biomarker for oral cancer detection. Nonetheless, further research is essential to validate these results.

## Figures and Tables

**Table 1:** Summary of GEO datasets for the analysis.

Study	Accession Number Dataset	Platform	Sample Size	Differential Expression Analysis	Tissue
Study_1	GSE125866 (6)	Illumina HiSeq 2100	8 oral submucous fibrosis (F) 8 oral cancer (C) 2 non-pathology (N)	F vs N C vs N	Oral cavity tissue
Study_2	GSE20116 (7)	AB SOLiD System 3.0	3 oral cancer (C)3 non-pathology (N)	C vs N	Oral cavity tissue
Study_3	GSE131568 (8)	Illumina HiSeq 4000	6 leukoplakia (L)6 non-pathology (N)	L vs N	Oral cavity tissue

## Data Availability

The data set generated during the current study are available from the Correspondence on reasonable request.
